# GIGANTEA gates gibberellin signaling through stabilization of the DELLA proteins in *Arabidopsis*

**DOI:** 10.1073/pnas.1913532116

**Published:** 2019-10-09

**Authors:** Maria A. Nohales, Steve A. Kay

**Affiliations:** ^a^Keck School of Medicine, University of Southern California, Los Angeles, CA 90089

**Keywords:** circadian clock, output oscillations, gating, GIGANTEA, gibberellin signaling

## Abstract

Circadian clock circuitry intersects with a plethora of signaling pathways to adequately time physiological processes to occur at the most appropriate time of the day and year. However, our mechanistic understanding of how the clockwork is wired to its output is limited. Here we uncover mechanistic connections between the core clock component GIGANTEA (GI) and hormone signaling through the modulation of key components of the transduction pathways. Specifically, we show how GI modulates gibberellin (GA) signaling through the stabilization of the DELLA proteins, which act as negative components in the signaling of this hormone. GI function within the GA pathway is required to precisely time the permissive gating of GA sensitivity, thereby determining the phase of GA-regulated physiological outputs.

## Significance

The circadian clock integrates environmental cues with internal biological processes to generate robust rhythms in almost all aspects of plant physiology. The molecular mechanisms underlying the pervasive regulation of plant physiology and development by the circadian clock are still being unraveled. Our study identifies the clock protein GIGANTEA as a key regulator of the response to gibberellins through the regulation of pivotal factors in the signaling of this hormone. Direct modulation of hub components in signaling networks by the circadian clock provides a means through which the oscillator can effectively transduce timing information to an extensive array of physiological pathways.

The circadian clock is an endogenous timekeeping molecular network that generates ∼24-h rhythms in myriad metabolic and physiological processes (1, 2). By providing time-of-day information, it allows plants to synchronize their endogenous physiology in anticipation of daily and seasonal fluctuations in environmental conditions, thereby enhancing fitness. Adequate coordination of output responses requires the intersection of the circadian circuitry with a multiplicity of other signaling pathways, giving rise to highly intricate regulatory systems. A key regulatory network with which the circadian oscillator intersects is hormone signaling. Phytohormones are signaling molecules that play a pivotal role in intrinsic plant developmental programs, as well as in the modulation of such programs in response to environmental cues, including biotic and abiotic stresses (3–5).

Both clock and hormone pathways are indispensable for the response to short- and long-term environmental challenges, and hence adequate crosstalk between them is crucial for plant adaptation to the local habitat. Accordingly, multiple connections between the oscillator and hormones exist (6). Specific hormones have been shown to affect circadian function (7), and, conversely, levels of many hormones display diel oscillatory patterns of accumulation, which are often circadian (8–11). These rhythms likely arise, at least partially, from transcriptional regulation by the clock, which broadly regulates the expression of hormone biosynthetic genes and signaling components (12–17).

A key hormone for plant growth and development is gibberellin (GA). GA is essential for numerous developmental processes, including seed germination, stem elongation, and flowering (18), many of which are also clock-regulated. The connection between GA and the central oscillator, however, appears to be asymmetric, where GAs operate as an output module within the circadian network that does not seem to feed back to the clock (7). In contrast, the clock directly influences GA levels and GA-regulated processes by affecting the transcription of genes involved in the biosynthesis and catabolism of GAs (17, 19). In a more elaborate mechanism, it has been proposed that the clock also gates the responsiveness to GAs by controlling the expression of the GA receptor gene *GA INSENSITIVE DWARF 1* (*GID1*) (16).

Because many core clock proteins are transcription factors, transcriptional regulation is a major mechanism through which the circadian clock delivers time information to output networks. However, it is becoming evident that, in order to robustly coordinate and fine-tune the outcome of such intricate regulatory webs, posttranscriptional connections are necessary. Here, we uncover direct protein–protein interactions between the core clock protein GIGANTEA (GI) and the DELLA proteins, negative components of GA signaling, that act as a key connection for clock output regulation. GI affects GA signaling through the stabilization of the DELLAs and is required to precisely time the gating of GA sensitivity to the early night, ultimately affecting the rhythmicity of physiological outputs such as hypocotyl elongation.

## Results and Discussion

### GI Interacts with the DELLA Proteins.

**Fig. 1.**
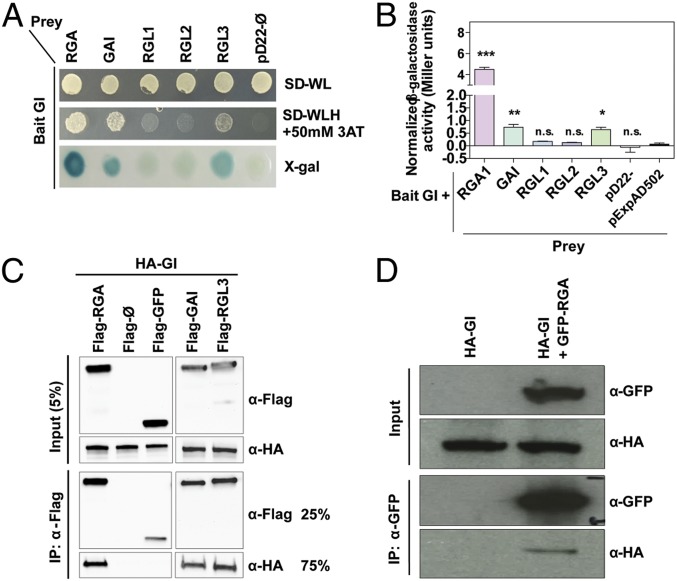
GI interacts with the DELLA proteins. (*A* and *B*) Yeast 2-hybrid (Y2H) assays showing interaction of GI and DELLA proteins. Bait and prey constructs were cotransformed into yeast cells. SD-WL, minimal medium lacking Trp and Leu; SD-WLH, selective medium lacking Trp, Leu, and His, which was supplemented with 50 mM 3AT. X-gal, qualitative β-galactosidase activity results obtained from the X-gal assay. (*B*) Quantitation of β-galactosidase activity (Miller units) for every pair of bait and prey proteins indicated (*n* = 4). Values represent means ± SEM [***P < 0.001; **P < 0.01; *P < 0.05; n.s., not significant Tukey’s multiple-comparison test relative to the pExpAD502 control vector]. (*C*) In vitro pull-down assays showing the interaction between GI and DELLAs (RGA, GAI, and RGL3). Proteins were expressed in an in vitro transcription and translation system. (*D*) In vivo coimmunoprecipitations in *Arabidopsis* transgenic seedlings expressing HA-GI (from the 35S promoter) and GFP–RGA (from an endogenous promoter fragment) tagged protein versions.

GI is a component of the circadian oscillator that is implicated in a plethora of biological processes (20). Although its precise molecular function still remains to be elucidated, it is well documented that GI is able to interact with and modulate the function of multiple proteins (20). In a yeast 2-hybrid (Y2H) assay performed to probe GI’s interaction network, we detected interaction of GI with the DELLA proteins REPRESSOR OF GA1-3 (RGA), GIBBERELLIC ACID INSENSITIVE (GAI), and RGA-LIKE PROTEIN 3 (RGL3) (Fig. 1 *A* and *B*). The DELLA proteins are a set of GRAS transcription regulators that function as negative components of GA signaling and repress GA-responsive genes, including growth-promoting genes (21, 22). DELLAs are proposed to function as hubs in plant development and physiology because they interact with multiple transcription factors from diverse pathways to regulate their activity, hence mediating crosstalk between GA and other signaling pathways (23, 24). Given the subtle effect that GAs have on circadian function (7), we considered unlikely that DELLAs affected GI activity and hence hypothesized that the interaction could entail the modulation of DELLA function by GI. Our Y2H results therefore raised the possibility that a mechanism underpinning GI’s widespread regulation of plant physiology involved the modulation of GA regulatory networks through interaction with the DELLA factors. We confirmed the observed interactions through in vitro pull-down assays with tagged full-length proteins expressed in an in vitro transcription and translation system (Fig. 1*C*). In the case of RGA, mapping of the interaction domains using defined protein fragments (*SI Appendix*, Fig. S1 *A* and *B*) revealed that all partial fragments except for the DELLA domain where able to interact with GI, likely because these fragments contain elements of the region mediating protein–protein interactions at its GRAS domain (25). Finally, we validated the GI–RGA interaction in vivo by performing coimmunoprecipitation studies in transgenic *Arabidopsis thaliana* seedlings expressing tagged protein versions (Fig. 1*D*).

### GI Stabilizes DELLAs in the Context of Their GA-Dependent Degradation.

**Fig. 2.**
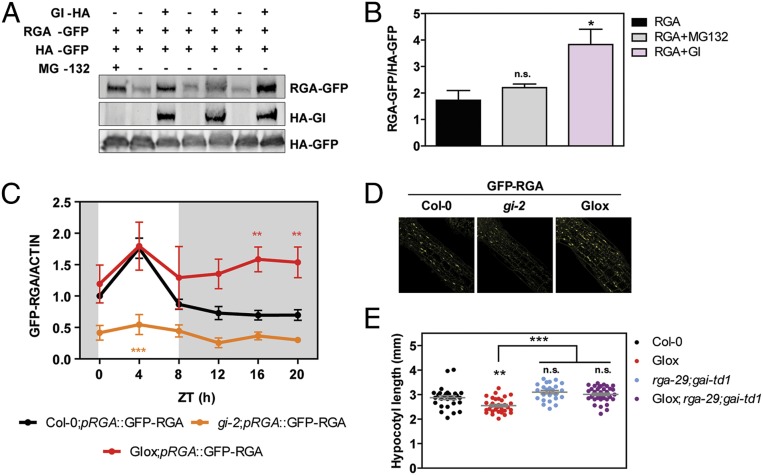
RGA is stabilized by GI, and GI function is required to shape oscillations in RGA protein accumulation. (*A*) Representative Western blot showing the accumulation of RGA-GFP in *N. benthamiana* leaves treated with 25 µM MG-132 or in the presence or absence of GI-HA. Protein levels were normalized against HA-GFP levels. (*B*) Quantitation of 3 biological replicates of the experiment shown in *A* (mean ± SEM; *P < 0.05; n.s., not significant Tukey’s multiple comparison test). Protein levels were normalized against HA-GFP levels. (*C*) Accumulation of GFP–RGA across SD photo-cycles in WT (Col-0), *gi-2*, and GIox backgrounds. ACTIN levels were used for normalization, and the quantitation of 3 biological replicates is shown (mean ± SEM; ***P < 0.001, **P < 0.01 Bonferroni post hoc test following 2-way ANOVA). White and gray shadings represent day and night, respectively. (*D*) Representative confocal images of 10-d-old SD-grown seedlings expressing GFP–RGA in WT (Col-0), *gi-2*, and GIox backgrounds taken from the upper part of the hypocotyl at ZT12. (*E*) Hypocotyl length measurements from WT (Col-0), GIox, *rga-29;gai-td1*, and GIox*;rga-29;gai-td1* seedlings grown for 7 d in SDs (mean ± SEM, *n* = 24 to 36; ***P < 0.001; **P < 0.01; n.s., not significant Tukey’s multiple comparison test).

Because several pathways in which GI functions involve the control of protein stability (26–28), and both GI and RGA accumulate as the day progresses (16, 29), we wondered if GI interaction with RGA could be contributing to RGA balance. At the transcriptional level, no major perturbations in *RGA* and *GAI* expression were observed in GI overexpression lines (GIox) (29) and *gi-2* mutant lines compared to wild-type (WT) plants (*SI Appendix*, Fig. S2*A*). Protein stability analyses of transient expression in *Nicotiana benthamiana* leaves revealed that RGA–GFP protein levels are indeed stabilized in the presence of GI (Fig. 2 *A* and *B*). To further investigate how GI contributes to shape diel RGA protein levels in vivo, we crossed an *Arabidopsis* transgenic line expressing GFP–RGA driven by an endogenous promoter fragment (30, 31) into the *gi-2* and GIox backgrounds. Western blot analysis of the protein levels in these lines across a 24-h cycle in short-day (SD) conditions confirmed that GI is required for the rhythmic pattern of RGA accumulation. RGA levels remained high even during the night phase when GI is overexpressed, whereas they were abrogated and low throughout the entire day in its absence (Fig. 2*C* and *SI Appendix*, Fig. S2*B*). Thus the presence of GI is required during the day to enable RGA accumulation at this time, and the absence of GI at night is necessary for RGA levels to decline. Inspection of the GFP–RGA transgenic lines in the different backgrounds under the confocal microscope revealed that strong differences in GFP–RGA accumulation in fact could be observed in the upper part of hypocotyls, which is the growing region (Fig. 2*D*). In line with these findings, loss of *RGA* and *GAI* was observed to alleviate the short hypocotyl phenotype of GIox lines (Fig. 2*E*), demonstrating that higher DELLA activity contributes to the restricted growth phenotype in GI overexpression lines.

**Fig. 3.**
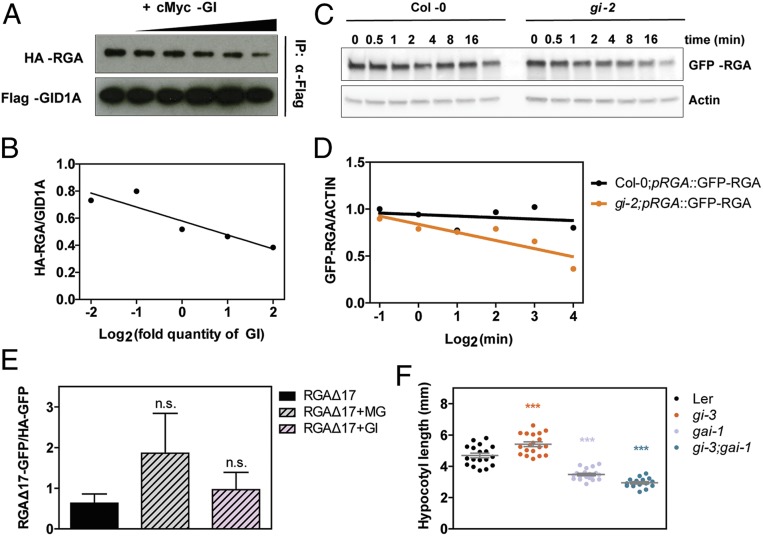
GI stabilizes RGA in the context of its GA-GID1–mediated degradation. (*A*) Interaction between Flag-GID1A and HA-RGA in the presence of increasing quantities of cMyc-GI (0.25×, 0.5×, 1×, 2×, and 4×). Proteins were expressed in a TnT in vitro expression system and immunoprecipitated with anti-Flag antibody. (*B*) Quantitation of the relative amount of HA—RGA coimmunoprecipitated with GID1A in every fraction from the experiment shown in *A*. (*C*) Degradation time course of GFP–RGA in WT (Col-0) and *gi-2* mutants. The 10-d-old SD-grown seedlings were treated at ZT7 with 100 µM GA_3_ and 200 µg/mL cyclohexamide. ACTIN levels were used for normalization. (*D*) Quantitation of the relative amount of GFP–RGA in every fraction from the experiment shown in *C*. Protein levels were normalized against ACTIN levels. (*E*) Quantitation of RGAΔ17-GFP accumulation in *N. benthamiana* leaves treated with 25 µM MG-132 or in the presence of GI–HA. Protein levels were normalized against HA–GFP levels. Values represent mean ± SEM (*n* = 3) (n.s., not significant Tukey’s multiple comparison test). (*F*) Hypocotyl length measurements from WT (Ler), *gi-3*, *gai-1*, and *gi-3;gai-1* seedlings grown for 7 d in SDs (in gray, mean ± SEM, *n* = 16 to 20; ***P < 0.001 Tukey’s multiple comparison test).

At the mechanistic level, we hypothesized that GI binding to RGA could hinder access of the GA receptor GID1 to RGA protein, thereby interfering with its degradation. Upon GA perception, the GID1 receptor undergoes a conformational change that increases its affinity for the DELLA proteins and promotes binding to them through their DELLA domain, which leads to their subsequent polyubiquitination and degradation by the 26S proteasome (22, 32). In vitro pull-down studies of GID1A-RGA binding in the absence and presence of GI confirmed that GI negatively affects this interaction (*SI Appendix*, Fig. S3*A*); additionally, increasing quantities of GI progressively decreased the amount of RGA coimmunoprecipitated with GID1A (Fig. 3 *A* and *B*). To explore the relevance of these findings in vivo, we performed GFP–RGA degradation time-course experiments in SD-grown *Arabidopsis* seedlings treated with GA at Zeitgeber time (ZT) 7. These experiments showed that GFP–RGA degrades faster in *gi-2* mutants compared to WT plants when treated with both GA_3_ and GA_4_ (Fig. 3 *C* and *D* and *SI Appendix*, Fig. S3*B*), and this difference is abolished in the presence of MG-132 (*SI Appendix*, Fig. S3 *C* and *D*). It is noteworthy that the altered stability of GFP–RGA likely reflects the actual behavior of the endogenous RGA because plants expressing the fusion protein in the *gi-2* background display long hypocotyls similar to those without the transgene (*SI Appendix*, Fig. S3*E*). Altogether, our observations support the role of GI as a stabilizing partner of RGA that gates its sensitivity to degradation through the GID1 pathway. In accordance with this notion, accumulation of RGAΔ17, a mutated version of RGA lacking the DELLA domain required to interact with the GID1 receptor, was not affected by the presence or absence of GI in *N. benthamiana* leaves (Fig. 3*E* and *SI Appendix*, Fig. S3*F*), and the dominant mutant *gai-1*, which causes a lesion similar to RGAΔ17 in the DELLA GAI, fully suppressed the long hypocotyl of *gi* mutants (Fig. 3*F*).

### GI Is Involved in the Circadian Gating of GA Signaling.

**Fig. 4.**
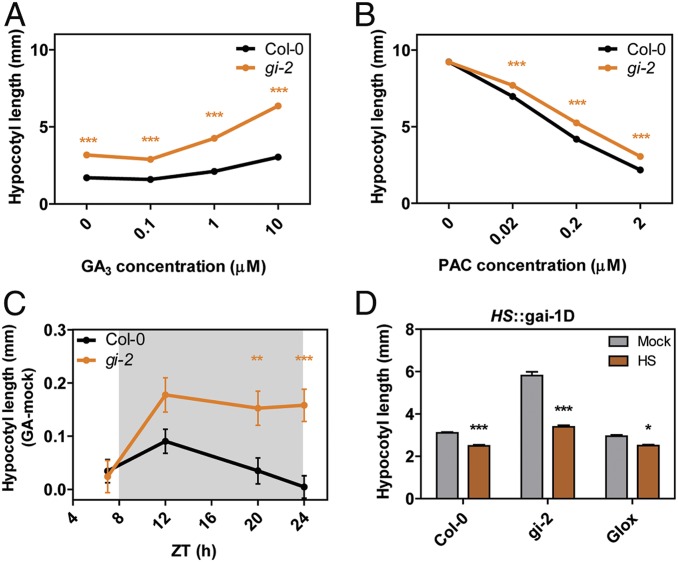
GI is required to adequately gate GA signaling at night. (*A* and *B*) GA_3_ and PAC dose–response curves for WT (Col-0) and *gi-2* mutant seedlings. Plants were grown for 7 d under SD conditions with increasing concentrations of GA_3_ (0, 0.1, 1, and 10 µM) (*A*) or for 3 d in the dark in the presence of increasing concentrations of PAC (0, 0.02, 0.2, and 2 µM) (*B*). Values represent means ± SEM (*n* = 24 to 36) (***P < 0.001; n.s., not significant Bonferroni post hoc test following 2-way ANOVA). (*C*) Hypocotyl length (measured as the difference between GA-treated and mock-treated seedlings) of seedlings grown for 6 d under SD conditions in the presence of 0.2 µM PAC and treated with 1 µM GA_4_ at different ZTs (mean ± SEM, *n* = 25) (n.s., not significant; **P < 0.01; ***P < 0.001 Bonferroni post hoc test following 2-way ANOVA). (*D*) Hypocotyl length of *HS*::gai-1D lines in WT (Col-0), *gi-2*, and GIox backgrounds treated with heat at ZT12 to induce the expression of the GAI dominant negative version *gai-1D* (brown bars) compared to nontreated controls (gray bars). Values represent mean ± SEM (*n* = 33 to 39) (*P < 0.05; ***P < 0.001 Tukey’s multiple comparison test). HS, heat-shock–treated plants.

Given that DELLAs are negative regulators of GA signaling (21, 22), RGA imbalance in *gi-2* mutants is expected to affect signaling of this hormone. Consistent with this notion, a dose–response curve in the presence of GA_3_ and the inhibitor of GA synthesis paclobutrazol (PAC) showed that *gi-2* mutants have indeed altered GA signaling, being hypersensitive to GA_3_ and hyposensitive to PAC (Fig. 4 *A* and *B*). Although it may be counterintuitive that *gi-2* plants are hypersensitive to GAs, it has to be considered that these mutants behave like a DELLA knockdown (as opposed to a knockout). These data suggest that the GA response is not fully derepressed, but rather less tightly repressed, and can therefore be more easily triggered compared to WT controls.

It has been proposed that the circadian gating of GA signaling arises from transcriptional regulation of the GA receptors by the clock, which results in higher stability of DELLA proteins during the day and higher sensitivity to GA at night (16). The mechanism underlying circadian transcriptional regulation of *GID1* is still unknown. Interestingly, GI binds the promoter region of *GID1A* (28), and *GID1A* expression is dysregulated in *gi-2* (33). Analysis of the expression of *GID1A* across a SD photocycle in *gi-2* mutants revealed that *GID1A* is induced in the middle of the night and at dawn (ZT16 and 24) (*SI Appendix*, Fig. S4*A*). However, its expression remained rhythmic and was not affected in the early night or by GI overexpression. Hence, although it is possible that GI additionally affects GA signaling through transcriptional regulation of the GA receptors, further more relevant regulators must exist.

In terms of phase, oscillations in *GID1* expression are also rather broad, with peak expression times spanning across most of the day. Transcript levels of *GID1A*, for example, are already high during daytime, when DELLAs accumulate, and remain high until the end of the night (16). This suggests that additional mechanisms exist that contribute to more precisely set the timing of GA sensitivity to the early night. We hence wondered if modulation of DELLA stability by GI may contribute to fine-tune the gating. To test this, we treated WT and *gi-2* plants with GA_4_ at different times of the day and measured the effect of this treatment on hypocotyl elongation compared to mock treatments. Treatment at ZT12 had the strongest effect in WT plants. In contrast, *gi-2* mutants showed a greater response at ZT12 and were responsive to the treatment across the entire night period regardless of the time of application (Fig. 4*C* and *SI Appendix*, Fig. S4*B*), suggesting that GI plays a role in the gating of this process. In terms of timing, the greater response to GAs that *gi* mutants display during the early night, which corresponds to the most sensitive period in WT plants, can be attributed to the lower DELLA levels in these lines, which translate into less tightly repressed GA-responsive genes. Considering the DELLA-stabilizing effect of GI, our results imply that GI at least partially gates GA sensitivity at night through the modulation of DELLA susceptibility to degradation. Further supporting the DELLA dependence of this phenotype, induction of expression of a dominant negative version of GAI during the night (ZT12) strongly suppressed the long hypocotyl phenotype of *gi-2* mutants (Fig. 4*D*). Nevertheless, it is still possible that GI additionally functions to repress the expression of GA-responsive genes more directly at this time, considering recent evidence on the function of GI in the repression of growth-promoting genes during the early night (28). Furthermore, GI also regulates *CCA1* and *LHY* transcription and consequently affects the expression of their target genes, which are expressed in the evening and are involved in different pathways including the response to GAs (28). At dawn, however, the role of GI in restricting GA sensitivity is most likely to arise only from indirect connections, given that GI levels are minimal at this time. One such plausible link are the PHYTOCHROME INTERACTING FACTORS (PIFs), as GI affects PIF activity and the expression of PIF target genes at dawn, and PIFs regulate the expression of GA biosynthetic genes (34). Another connection between GI and GA signaling that must be considered is SPINDLY (SPY). GI interacts with SPY in vitro (35), and SPY has recently been shown to *O*-fucosylate the DELLAs, thereby activating them and promoting their interaction with key regulators in brassinosteroid- and light-signaling pathways, including BRASSINAZOLE-RESISTANT1 (BZR1), PIF3, and PIF4 (36). It would therefore be interesting to investigate whether the GI–SPY interaction has any implications for DELLA *O*-fucosylation, if SPY is involved in GI-mediated stabilization of the DELLAs, and what the connection might be to the repression of PIF activity.

Hence GI seems to function in intricate ways to pervasively regulate output physiology (such as photoperiodic growth), not only by modulating the light signaling pathway as recently demonstrated (28), but also by fine-tuning the response to GAs through several complementary mechanisms.

## Concluding Remarks

The circadian oscillator plays a pivotal role in the integration of external cues with endogenous physiology, orchestrating plant physiological processes to occur at the most advantageous time of the day and year. This function contributes to optimize resource allocation and hence enhances fitness (37). To adequately coordinate output processes, the biological clock intersects with a wide array of other signaling pathways, rendering highly intricate regulatory networks. Such complexity is likely to pose an advantage for coping with the wide range of environmental changes and challenges that plants are exposed to, as the existence of multiple checkpoints increases both the flexibility and robustness of the system.

**Fig. 5.**
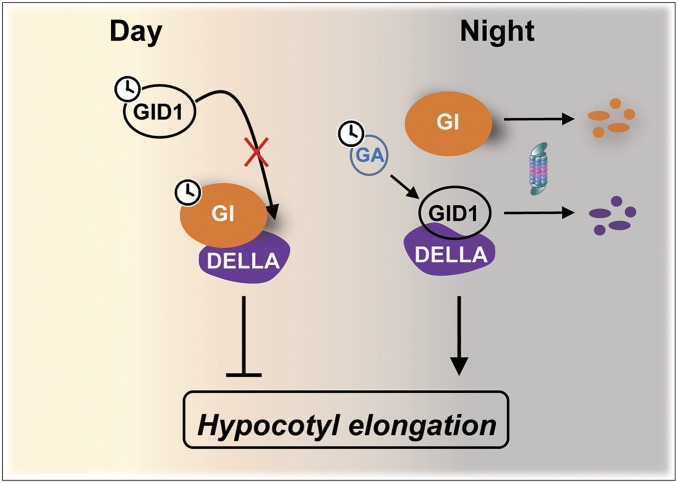
Model of GI action in the gating of GA signaling. As GI accumulates during the day, it stabilizes the DELLAs by hindering access of the GA receptor GID1A, the expression of which is circadian-regulated and high in the evening. Progressive degradation of GI during the evening enables the degradation of the DELLA proteins and the expression of GA-responsive genes, including growth-promoting genes.

Here we provide insights into a mechanism linking circadian timing to hormone signaling and uncover GI as a core clock component involved in the gating of the response to GAs (Fig. 5). GI interacts with the negative components of GA signaling, the DELLA factors, and stabilizes them in the context of their GA-mediated degradation. This directly influences the rhythmic pattern of DELLA accumulation across the day and the timing of maximal sensitivity to GAs. While a connection between daily rhythms in GA-signaling components and the gating of the sensitivity to this hormone has been previously proposed (16), mechanistic connections to the oscillatory machinery were missing. Hence, our work uncovers an important mechanism by which circadian phasing and endogenous hormone signaling pathways are integrated.

## Materials and Methods

### Plant Material and Growth Conditions.

WT, mutant, and transgenic lines used in this study were *A. thaliana* ecotype Columbia 0 (Col-0) with the exception of the *gi-3* and *gai-1* mutants, which were ecotype Landsberg *erecta* (L*er*). *gi-2* (38), GIox (29), pRGA::GFP-RGA (30, 31), *rga-29* (SALK_89146) (39, 40), *gai-td1* (SAIL_82_F06) (41), *gi-3* (42), *gai-1* (43), and *HS*::gai-1D (44) have been previously described. *gi-3* lines were obtained from the *Arabidopsis* Biological Research Center collection. Seeds were chlorine-gas-sterilized and plated on 0.5× Linsmaier and Skoog (LS) medium (Caisson Laboratories) with 0.8% agar (Sigma). After stratification in the dark at 4 °C for 3 d, plates were transferred to a Percival incubator (Percival Scientific) set to the indicated light conditions with light supplied at 80 μmol⋅m^−2^⋅s^−1^ by cool-white fluorescent bulbs and a constant temperature of 22 °C.

### Generation of Higher Order Mutants and Transgenic Lines.

*pRGA*::GFP-RGA;GIox and *pRGA*::GFP-RGA;*gi-2* lines were obtained by crossing *pRGA*::GFP-RGA (kanamycin resistance) with GIox plants (BASTA resistant and *gi-2*). Similarly, *HS*::gai-1D;GIox and *HS*::gai-1D;*gi-2* lines were obtained by crossing *HS*::gai-1D (kanamycin resistance) with GIox plants. F3 populations were screened for kanamycin resistance and BASTA resistance/sensitivity. The presence of the *gi-2* allele in all lines was determined by PCR amplification with primers listed in Table S1.

The GIox;*rga-29*;*gai-td1* triple mutant was obtained by crossing GIox into the *rga-29;gai-td1* double mutant. F3 homozygous BASTA-resistant descendants were screened for the presence of the *rga-29* and *gai-td1* alleles by PCR amplification with primers listed in Table S1. Since the presence of a homozygous *gi-2* mutation in the *rga-29;gai-td1* mutant resulted in lethality, a GIox;Col-0 line was also obtained from this cross and used as a control line for hypocotyl measurements.

*gi-3;gai-1* mutants were generated by genetic crosses between the single mutants, and homozygous mutant lines were identified in the F2 populations by PCR amplification with primers listed in Table S1. The *gi-3* allele was identified by its late flowering phenotype

### Construction of Binary Vectors.

To perform protein stability assays in transient expression in *N. benthamiana*, the cDNAS encoding GI, RGA, RGAΔ17, and GFP were amplified by PCR from their respective pENTR/D-TOPO vectors (45) (primers used are listed in Table S1) and subcloned into the pDONR207 vector (Invitrogen) by Gateway BP recombination reaction (Invitrogen). Subsequently, the coding sequences were transferred to an array of pEarleyGate (46) (pEG) binary destination vectors by Gateway LR recombination reaction (Invitrogen). The sequences introduced into the pEG201 plasmid contained stop codon, while the ones introduced into the pEG103 plasmid did not. Specifically, the different constructs generated and used were as follows: pEG103-RGA, pEG103- RGAΔ17, pEG201-GFP, and pEG201-GI.

### Yeast Two-Hybrid Analyses.

We used the ProQuest Two-Hybrid System (Invitrogen) as previously described (28). Specifically, the cDNA encoding full-length GI was transferred from the pENTR/D-TOPO vector (Invitrogen) into the pDEST32 vector by Gateway LR recombination reaction (Invitrogen) to generate the bait plasmid; the pDEST22 prey plasmids containing the sequences encoding RGA, GAI, RGL1, RGL2, and RGL3 have been previously described (45). Empty pDEST22 and the pExpAD502 plasmids were used as negative controls. Quantitation of β-galactosidase activity was performed in a 96-well format as previously described (47).

### In Vitro Pull-Downs.

For in vitro pull-down assays, additional constructs were made. The pENTR/D-TOPO plasmid (Invitrogen) containing the sequence encoding GI has been previously described (45, 48). Full-length *RGA*, *GAI*, *RGL3*, *GID1A*, and *GFP*, as well as partial *RGA* sequences, were amplified by PCR (primers used are listed in Table S1) and cloned into the pENTR/D-TOPO vector (Invitrogen). The pENTR-RGA plasmid was used to create the pENTR-RGAΔ17 truncated version by PCR-based mutagenesis using the primers listed in Table S1. To express proteins in the cell-free system, all inserts were transferred by Gateway LR recombination reaction (Invitrogen) into Gateway-compatible modified pTnT vectors (Promega) (49), which were kindly provided by Joanne Chory (The SALK Institute, La Jolla, CA). The vectors contained an N-terminal HA, Flag, or cMyc tag as specified in each case. Proteins were coexpressed using the TnT SP6 High-Yield Wheat Germ Protein Expression System (Promega) per manufacturer’s instructions. Five percent of the reactions (2.5 µL) were used to verify expression of the proteins (input), and the remaining extract was immunoprecipitated as described earlier (50) using either anti-Flag M2 (Sigma) or anti-HA 3F10 (Roche) antibodies, as specified in each case. Exceptionally for the analysis of the GID1A–RGA interaction in the presence and absence of GI, the proteins were expressed separately and 5% of the reactions (2.5 µL) was used to verify expression of the proteins (input). Seven microliters of each GID1A and RGA extract were combined, and 0, 1.75, 3.5, 7, 15, or 28 µL of the GI extract were added. For immunoprecipitation, we verified that the addition of GA_3_ to the immunoprecipitation (IP) buffer did not promote degradation of RGA, and 100 µM GA_3_ was added to the IP buffer to promote the interaction between GID1A and RGA.

### Transient Expression in *N. benthamiana*.

Experiments were performed as previously described (28). Briefly, *Agrobacterium tumefaciens* strain GV3101 cells containing the respective constructs and the p19 silencing suppressor were grown overnight at 28 °C in liquid Luria–Bertani medium supplemented with the appropriate antibiotics. Cultures were pelleted, resuspended in 10 mM MES–KOH, pH 5.6, 10 mM MgCl_2,_ 150 µM acetosyringone to a final OD_600_ of 0.5 and incubated for 2 h at room temperature. The suspensions were then mixed and infiltrated in *N. benthamiana* leaves at a final OD_600_ of 0.1 each, except for p19, which was infiltrated at a final OD_600_ of 0.05. Plants were kept in the greenhouse (under long-day conditions), and samples were harvested 3 d post inoculation. For MG-132 treatments, leaves were infiltrated with 10 mM MES–KOH, pH 5.6, 10 mM MgCl2, and 25 µM MG-132 at least 8 h prior to harvesting.

### Protein Immunoprecipitation.

Protein immunoprecipitations were performed as previously described (28). Approximately 1 g of 10-d-old *Arabidopsis* seedlings grown in SDs was harvested at ZT8 and frozen in liquid nitrogen. Immunoprecipitations were performed as earlier described (51) with the following modifications. Samples were ground with mortar and pestle in liquid nitrogen and resuspended in 2 mL of modified SII buffer [100 mM Na/phosphate, pH 8.0, 150 mM NaCl, 5 mM ethylenediamine tetraacetic acid, 5 mM egtazic acid, 0.1% Triton X-100, 2 mM phenylmethylsulfonyl fluoride, 1× protease inhibitor mixture (Roche), 1× Phosphatase Inhibitors I and II (Sigma), and 50 μM MG-132 (Peptides International)]. Extracts were transferred to a dounce tissue grinder and homogenized before being clarified twice by centrifugation at 4 °C. Total protein concentration was quantified by DC Protein Assay (Bio-Rad) and normalized to 1.875 mg/mL. Three percent of the extracts was used to verify proteins levels (input). For immunoprecipitations, extracts were incubated with anti-GFP Ab290 (Abcam) antibody for 2 h with gentle rotation at 4 °C. Subsequently, 25 µL of magnetic protein G Dynabeads (Invitrogen) prewashed with IP buffer were added to the samples and incubated for 2 h with gentle rotation at 4 °C. The samples were finally washed 3× with modified SII buffer, and the precipitated protein was eluted by heating beads at 95 °C for 5 min in 40 μL of 2× sodium dodecyl sulfate polyacrylamide gel electrophoresis (SDS-PAGE) loading buffer. A total of 10 and 30 µL of the eluate were separately analyzed by Western blot to detect the immunoprecipitated and coimmunoprecipitated proteins, respectively.

### Protein Stability and Accumulation Analyses.

To determine protein levels in transient experiments in *N. benthamiana* samples were harvested at ZT12 and frozen in liquid nitrogen. They were then ground and homogenized with 3 volumes of 2× SDS-PAGE loading buffer and boiled at 95 °C for 5 min. Samples were then clarified by centrifugation at room temperature and analyzed by Western blot. For normalization, GFP-HA was used as internal loading control.

Time-course degradation experiments of GFP-RGA in *Arabidopsis* seedlings were performed as follows. Approximately 40 10-d-old SD-grown *Arabidopsis* seedlings per sample were transferred at ZT7 to 2-mL transparent tubes and incubated in the light for 0.5, 1, 2, 4, 8, or 16 min in liquid 0.5× LS medium in the presence of 100 µM GA_3_ (Sigma) and 200 µg/mL cyclohexamide (Sigma) or 50 µM MG-132, 100 µM GA_3_ (Sigma), and 200 µg/mL cyclohexamide (Sigma). At the specified time points, the seedlings were extracted from the tubes and rapidly placed on tissue paper to remove liquid excess before freezing them in liquid nitrogen. For the analysis of GFP–RGA protein levels in *Arabidopsis* seedlings, samples were homogenized with 100 µL of extraction buffer [50 mM Tris⋅HCl, pH 7.6, 150 mM NaCl, 5 mM MgCl_2_, 0.1% Nonidet P-40, 10% glycerol, 2 mM PMSF, 1× protease inhibitor mixture (Roche), 1× Phosphatase Inhibitors I and II (Sigma) and 50 μM MG-132 (Peptides International)] and clarified twice by centrifugation at 4 °C. In both cases, total protein concentration was quantified by DC Protein Assay (Bio-Rad), and 40 μg of each sample was subsequently analyzed by Western blot. ACTIN levels in the samples were used for normalization.

### Western Blot Detection and Quantitation.

The procedure was performed as previously described (28). Protein extracts in SDS-PAGE loading buffer were boiled at 95 °C for 5 min and separated in 4 to 15% SDS-PAGE gels. Proteins were then transferred to nitrocellulose membranes (Bio-Rad), which were then stained with Ponceau S to assess transfer and loading. Finally, membranes were immunodetected with either horseradish peroxidase (HRP)-conjugated 3F10 anti-HA (1:2,000, Roche), HRP-conjugated Flag M2 (1:2,000, Sigma), HRP-conjugated anti-cMyc (1:2,000, Invitrogen), or anti-GFP antibody (1:2,000, Roche) followed by HRP-conjugated anti-mouse secondary antibody (1:3,000, Bio-Rad). The ACTIN loading control was detected using anti-ACTIN C4 mouse antibody (1:500, Millipore) followed HRP-conjugated anti-mouse secondary antibody (1:3,000, Bio-Rad). For *Arabidopsis* GFP–RGA blots, the membrane was cut above the 75-kDa mark, and the upper and lower parts were detected with anti-GFP and anti-ACTIN antibodies, respectively. Chemiluminescence was detected with the Supersignal West Pico, Dura, and Femto substrates (Pierce) and imaged with a UVP ChemiDoc imaging system. The VisionWorksLS (UVP) software was used to quantify protein levels. In the case of the analysis of the interaction between GID1A and RGA in the presence and absence of GI, chemiluminescence was captured by exposure of X-ray films (GE Healthcare), which were scanned, and protein levels were quantified using NIH ImageJ software (https://imagej.nih.gov/ij/).

### RNA Extraction and qRT-PCR.

As previously described (28), total RNA was isolated with the GeneJET plant RNA purification mini kit (Thermo Scientific). For cDNA synthesis, 1 μg of total RNA was digested with DNase I (Roche) and reverse-transcribed using the iScript cDNA synthesis kit (Bio-Rad). Synthesized cDNA was amplified by real-time quantitative PCR (qPCR) with Maxima SYBR Green qPCR Master Mix (Thermo Scientific) using the CFX-384 Real Time System (Bio-Rad). *PROTEIN PHOSPHATASE 2A* (*PP2A*) (AT1G13320) was used as the normalization control. Primer sequences are listed in Table S1.

### Confocal Imaging.

Confocal microscopy was performed with a Zeiss LSM 710 laser-scanning confocal microscope (Carl Zeiss). GFP was excited with the excitation beam splitter MBS 488, and emission was measured with an emission filter set at 493 to 550 nm. Image analysis was performed with ZEN software (Carl Zeiss). Representative images from at least 2 independent biological repeats are shown in this study.

### Physiological Measurements.

To analyze hypocotyl length, evenly spaced seedlings were grown on plates under the light conditions and photoperiod indicated in the figures. At the specified time, seedlings were scanned, and images were analyzed using NIH ImageJ software (https://imagej.nih.gov/ij/).

GA sensitivity assays at different ZTs were performed as previously described (16) with minor modifications. Briefly, seedlings were grown on filter papers placed on 0.5× LS, 0.8% agar, 0.2 µM PAC plates for 3 d in SDs. On the fourth day, filter papers containing 4-d-old seedlings were transferred at ZT0, 7, 12, or 20 for 1 h to petri dishes with 5 mL of 0.5× LS liquid medium containing 0.1 μM GA_4_ (Sigma) and 0.2 μM PAC (GA treatment) or just 0.2 μM PAC (mock). After the treatment, the filter papers with the seedlings were rinsed 3 times for 20 min in petri dishes containing liquid 0.5× LS with PAC 0.2 μM. After the washes, seedlings were transferred to new sterile filter papers, placed on fresh 0.5× LS, 0.2 μM PAC plates, and returned to SD conditions. GA treatments were applied during 2 consecutive days (fourth and fifth days), and hypocotyl length was measured on day 6. Hypocotyl length increase upon GA_4_ treatment at the different ZTs was calculated as the difference between GA_4_-treated plants and mock-treated controls.

For heat-shock treatments to induce the expression of gai-1D, plates containing seedlings of the different genotypes grown in SDs were incubated at ZT12 for 10 min at 37 °C in darkness, while control seedlings were kept at 22 °C. Heat treatments were applied at days 3, 4, 5, and 6. Hypocotyl length was measured on day 7.

## Supplementary Material

Supplementary File

Supplementary File

## References

[1] Greenham K., McClung C. R., Integrating circadian dynamics with physiological processes in plants. Nat. Rev. Genet. 16, 598–610 (2015). 26370901 10.1038/nrg3976

[2] Sanchez S. E., Kay S. A., The plant circadian clock: From a simple timekeeper to a complex developmental manager. Cold Spring Harb. Perspect. Biol. 8, a027748 (2016). 27663772 10.1101/cshperspect.a027748 PMC5131769

[3] Ku Y. S., Sintaha M., Cheung M. Y., Lam H. M., Plant hormone signaling crosstalks between biotic and abiotic stress responses. Int. J. Mol. Sci. 19, E3206 (2018). 30336563 10.3390/ijms19103206 PMC6214094

[4] Eyidogan F., Oz M. T., Yucel M., Oktem H. A., “Signal transduction of phytohormones under abiotic stresses” in Phytohormones and Abiotic Stress Tolerance in Plants, Khan N., Nazar R., Iqbal N., Anjum N., Eds. (Springer, Berlin, 2012).

[5] Takatsuji H., Jiang C. J., “Plant hormone crosstalks under biotic stresses” in Phytohormones: A Window to Metabolism, Signaling and Biotechnological Applications, Tran L. S., Pal S., Eds. (Springer, New York, 2014).

[6] Atamian H. S., Harmer S. L., Circadian regulation of hormone signaling and plant physiology. Plant Mol. Biol. 91, 691–702 (2016). 27061301 10.1007/s11103-016-0477-4

[7] Hanano S., Domagalska M. A., Nagy F., Davis S. J., Multiple phytohormones influence distinct parameters of the plant circadian clock. Genes Cells 11, 1381–1392 (2006). 17121545 10.1111/j.1365-2443.2006.01026.x

[8] Thain S. C., Circadian rhythms of ethylene emission in Arabidopsis. Plant Physiol. 136, 3751–3761 (2004). 15516515 10.1104/pp.104.042523 PMC527172

[9] Bancos S., Diurnal regulation of the brassinosteroid-biosynthetic CPD gene in Arabidopsis. Plant Physiol. 141, 299–309 (2006). 16531479 10.1104/pp.106.079145 PMC1459315

[10] Covington M. F., Harmer S. L., The circadian clock regulates auxin signaling and responses in Arabidopsis. PLoS Biol. 5, e222 (2007). 17683202 10.1371/journal.pbio.0050222 PMC1939880

[11] Nováková M., Diurnal variation of cytokinin, auxin and abscisic acid levels in tobacco leaves. J. Exp. Bot. 56, 2877–2883 (2005). 16157652 10.1093/jxb/eri282

[12] Mizuno T., Yamashino T., Comparative transcriptome of diurnally oscillating genes and hormone-responsive genes in Arabidopsis thaliana: Insight into circadian clock-controlled daily responses to common ambient stresses in plants. Plant Cell Physiol. 49, 481–487 (2008). 18202002 10.1093/pcp/pcn008

[13] Shin J., Heidrich K., Sanchez-Villarreal A., Parker J. E., Davis S. J., TIME FOR COFFEE represses accumulation of the MYC2 transcription factor to provide time-of-day regulation of jasmonate signaling in Arabidopsis. Plant Cell 24, 2470–2482 (2012). 22693280 10.1105/tpc.111.095430 PMC3406923

[14] Wang G., Zhang C., Battle S., Lu H., The phosphate transporter PHT4;1 is a salicylic acid regulator likely controlled by the circadian clock protein CCA1. Front. Plant Sci. 5, 701 (2014). 25566276 10.3389/fpls.2014.00701 PMC4267192

[15] Zheng X. Y., Spatial and temporal regulation of biosynthesis of the plant immune signal salicylic acid. Proc. Natl. Acad. Sci. U.S.A. 112, 9166–9173 (2015). 26139525 10.1073/pnas.1511182112 PMC4522758

[16] Arana M. V., Marín-de la Rosa N., Maloof J. N., Blázquez M. A., Alabadí D., Circadian oscillation of gibberellin signaling in Arabidopsis. Proc. Natl. Acad. Sci. U.S.A. 108, 9292–9297 (2011). 21576475 10.1073/pnas.1101050108 PMC3107313

[17] Zhao X., A study of gibberellin homeostasis and cryptochrome-mediated blue light inhibition of hypocotyl elongation. Plant Physiol. 145, 106–118 (2007). 17644628 10.1104/pp.107.099838 PMC1976579

[18] Achard P., Genschik P., Releasing the brakes of plant growth: How GAs shutdown DELLA proteins. J. Exp. Bot. 60, 1085–1092 (2009). 19043067 10.1093/jxb/ern301

[19] Blázquez M. A., Trénor M., Weigel D., Independent control of gibberellin biosynthesis and flowering time by the circadian clock in Arabidopsis. Plant Physiol. 130, 1770–1775 (2002). 12481060 10.1104/pp.007625 PMC166688

[20] Mishra P., Panigrahi K. C., GIGANTEA: An emerging story. Front. Plant Sci. 6, 8 (2015). 25674098 10.3389/fpls.2015.00008 PMC4306306

[21] Hauvermale A. L., Ariizumi T., Steber C. M., Gibberellin signaling: A theme and variations on DELLA repression. Plant Physiol. 160, 83–92 (2012). 22843665 10.1104/pp.112.200956 PMC3440232

[22] Sun T. P., The molecular mechanism and evolution of the GA-GID1-DELLA signaling module in plants. Curr. Biol. 21, R338–R345 (2011). 21549956 10.1016/j.cub.2011.02.036

[23] Davière J. M., Achard P., A pivotal role of DELLAs in regulating multiple hormone signals. Mol. Plant 9, 10–20 (2016). 26415696 10.1016/j.molp.2015.09.011

[24] Xu H., Liu Q., Yao T., Fu X., Shedding light on integrative GA signaling. Curr. Opin. Plant Biol. 21, 89–95 (2014). 25061896 10.1016/j.pbi.2014.06.010

[25] Li S., Crystal structure of the GRAS domain of SCARECROW-LIKE7 in Oryza sativa. Plant Cell 28, 1025–1034 (2016). 27081181 10.1105/tpc.16.00018 PMC4904676

[26] Sawa M., Nusinow D. A., Kay S. A., Imaizumi T., FKF1 and GIGANTEA complex formation is required for day-length measurement in Arabidopsis. Science 318, 261–265 (2007). 17872410 10.1126/science.1146994 PMC3709017

[27] Kim W. Y., ZEITLUPE is a circadian photoreceptor stabilized by GIGANTEA in blue light. Nature 449, 356–360 (2007). 17704763 10.1038/nature06132

[28] Nohales M. A., Multi-level modulation of light signaling by GIGANTEA regulates both the output and pace of the circadian clock. Dev. Cell 49, 840–851.e8 (2019). 31105011 10.1016/j.devcel.2019.04.030 PMC6597437

[29] David K. M., Armbruster U., Tama N., Putterill J., Arabidopsis GIGANTEA protein is post-transcriptionally regulated by light and dark. FEBS Lett. 580, 1193–1197 (2006). 16457821 10.1016/j.febslet.2006.01.016

[30] Silverstone A. L., Repressing a repressor: Gibberellin-induced rapid reduction of the RGA protein in Arabidopsis. Plant Cell 13, 1555–1566 (2001). 11449051 10.1105/TPC.010047 PMC139546

[31] Shani E., Gibberellins accumulate in the elongating endodermal cells of Arabidopsis root. Proc. Natl. Acad. Sci. U.S.A. 110, 4834–4839 (2013). 23382232 10.1073/pnas.1300436110 PMC3606980

[32] Murase K., Hirano Y., Sun T. P., Hakoshima T., Gibberellin-induced DELLA recognition by the gibberellin receptor GID1. Nature 456, 459–463 (2008). 19037309 10.1038/nature07519

[33] Kim Y., GIGANTEA and EARLY FLOWERING 4 in Arabidopsis exhibit differential phase-specific genetic influences over a diurnal cycle. Mol. Plant 5, 678–687 (2012). 22328721 10.1093/mp/sss005 PMC3355345

[34] Filo J., Gibberellin driven growth in elf3 mutants requires PIF4 and PIF5. Plant Signal. Behav. 10, e992707 (2015). 25738547 10.4161/15592324.2014.992707 PMC4622946

[35] Tseng T. S., Salomé P. A., McClung C. R., Olszewski N. E., SPINDLY and GIGANTEA interact and act in Arabidopsis thaliana pathways involved in light responses, flowering, and rhythms in cotyledon movements. Plant Cell 16, 1550–1563 (2004). 15155885 10.1105/tpc.019224 PMC490045

[36] Zentella R., The Arabidopsis O-fucosyltransferase SPINDLY activates nuclear growth repressor DELLA. Nat. Chem. Biol. 13, 479–485 (2017). 28244988 10.1038/nchembio.2320 PMC5391292

[37] Millar A. J., The intracellular dynamics of circadian clocks reach for the light of ecology and evolution. Annu. Rev. Plant Biol. 67, 595–618 (2016). 26653934 10.1146/annurev-arplant-043014-115619

[38] Fowler S., GIGANTEA: A circadian clock-controlled gene that regulates photoperiodic flowering in Arabidopsis and encodes a protein with several possible membrane-spanning domains. EMBO J. 18, 4679–4688 (1999). 10469647 10.1093/emboj/18.17.4679 PMC1171541

[39] Tyler L., Della proteins and gibberellin-regulated seed germination and floral development in Arabidopsis. Plant Physiol. 135, 1008–1019 (2004). 15173565 10.1104/pp.104.039578 PMC514135

[40] Park J., Gibberellin signaling requires chromatin remodeler PICKLE to promote vegetative growth and phase transitions. Plant Physiol. 173, 1463–1474 (2017). 28057895 10.1104/pp.16.01471 PMC5291033

[41] Plackett A. R., DELLA activity is required for successful pollen development in the Columbia ecotype of Arabidopsis. New Phytol. 201, 825–836 (2014). 24400898 10.1111/nph.12571 PMC4291109

[42] Mizoguchi T., Distinct roles of GIGANTEA in promoting flowering and regulating circadian rhythms in Arabidopsis. Plant Cell 17, 2255–2270 (2005). 16006578 10.1105/tpc.105.033464 PMC1182487

[43] Dill A., Thomas S. G., Hu J., Steber C. M., Sun T. P., The Arabidopsis F-box protein SLEEPY1 targets gibberellin signaling repressors for gibberellin-induced degradation. Plant Cell 16, 1392–1405 (2004). 15155881 10.1105/tpc.020958 PMC490034

[44] Alabadí D, Gibberellins modulate light signaling pathways to prevent Arabidopsis seedling de-etiolation in darkness. Plant J. 53, 324–335 (2008). 18053005 10.1111/j.1365-313X.2007.03346.x

[45] Pruneda-Paz J. L., A genome-scale resource for the functional characterization of Arabidopsis transcription factors. Cell Rep. 8, 622–632 (2014). 25043187 10.1016/j.celrep.2014.06.033 PMC4125603

[46] Earley K. W., Gateway-compatible vectors for plant functional genomics and proteomics. Plant J. 45, 616–629 (2006). 16441352 10.1111/j.1365-313X.2005.02617.x

[47] Pruneda-Paz J. L., Breton G., Para A., Kay S. A., A functional genomics approach reveals CHE as a component of the Arabidopsis circadian clock. Science 323, 1481–1485 (2009). 19286557 10.1126/science.1167206 PMC4259050

[48] Sawa M., Kay S. A., GIGANTEA directly activates Flowering Locus T in Arabidopsis thaliana. Proc. Natl. Acad. Sci. U.S.A. 108, 11698–11703 (2011). 21709243 10.1073/pnas.1106771108 PMC3136272

[49] Nito K., Wong C. C., Yates J. R. III, Chory J., Tyrosine phosphorylation regulates the activity of phytochrome photoreceptors. Cell Rep. 3, 1970–1979 (2013). 23746445 10.1016/j.celrep.2013.05.006 PMC4023694

[50] Pedmale U. V., Cryptochromes interact directly with PIFs to control plant growth in limiting blue light. Cell 164, 233–245 (2016). 26724867 10.1016/j.cell.2015.12.018 PMC4721562

[51] Nusinow D. A., The ELF4-ELF3-LUX complex links the circadian clock to diurnal control of hypocotyl growth. Nature 475, 398–402 (2011). 21753751 10.1038/nature10182 PMC3155984

