# Supplementary material for: GIGANTEA gates gibberellin signaling through stabilization of the DELLA proteins in Arabidopsis
Source: Proc Natl Acad Sci U S A. 2019 Oct 9;116(43):21893–9. doi: 10.1073/pnas.1913532116 (PMC6815129; doi:10.1073/pnas.1913532116)
Supplement: Supplementary File [file pnas.1913532116.sapp.pdf]

PNAS
www.pnas.org

Supplementary Information for

GIGANTEA gates gibberellin signaling through stabilization of the DELLA proteins in *Arabidopsis*

Maria A. Nohales[a*] & Steve A. Kay[a*]

*Correspondence to:
Maria A. Nohales, E-mail: nohalesz@usc.edu
Steve A. Kay, E-mail: stevekay@usc.edu

This PDF file includes:

Fig. S1 to S4

Other supplementary materials for this manuscript include the following:

Table S1 Primers

www.pnas.org/cgi/doi/10.1073/pnas.1913532116

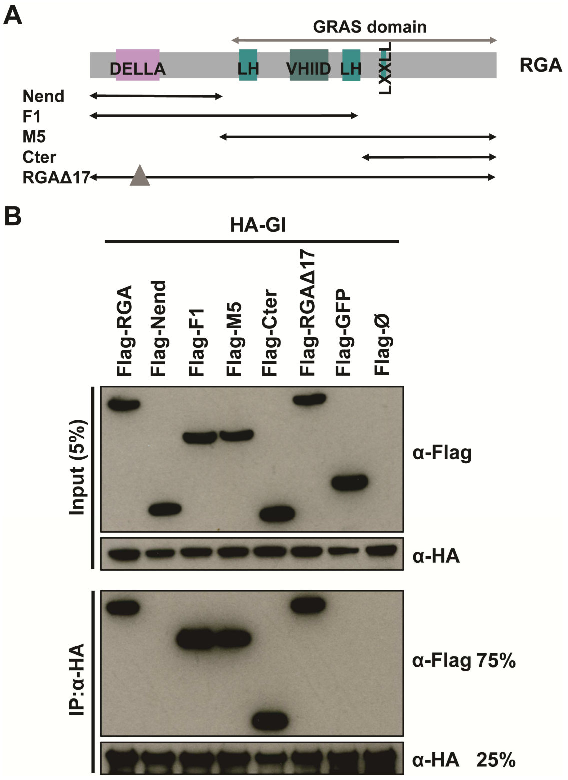

Fig. S1. GI interacts with the GRAS domain of RGA.

(*A*) Scheme of the deleted protein versions used to map the interaction domains between GI and RGA. (*B*) *In vitro* pull-down assays performed to map the interaction domains between GI and RGA. Proteins were expressed in a TnT *in vitro* expression system and immunoprecipitated with anti-HA antibody. The recovered fractions were analyzed by Western blot using anti-Flag and anti-HA antibodies.

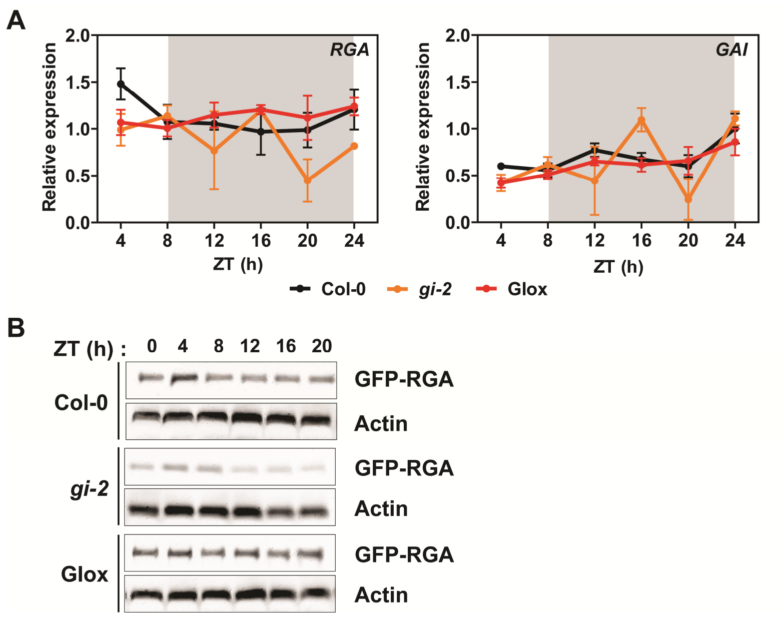

Fig. S2. GI function is required to shape oscillations in RGA protein accumulation (*A*) Relative expression of *RGA* and *GAI* in WT (Col-0), *gi-2*, and Glox across a SD photocycle in seedlings grown for 10 days in SDs (mean ± SEM of 3 biological replicates). White and gray shadings represent day and night, respectively. (*B*) Representative Western blot of the experiment shown in Fig. 2*C*. ACTIN levels were used for normalization.

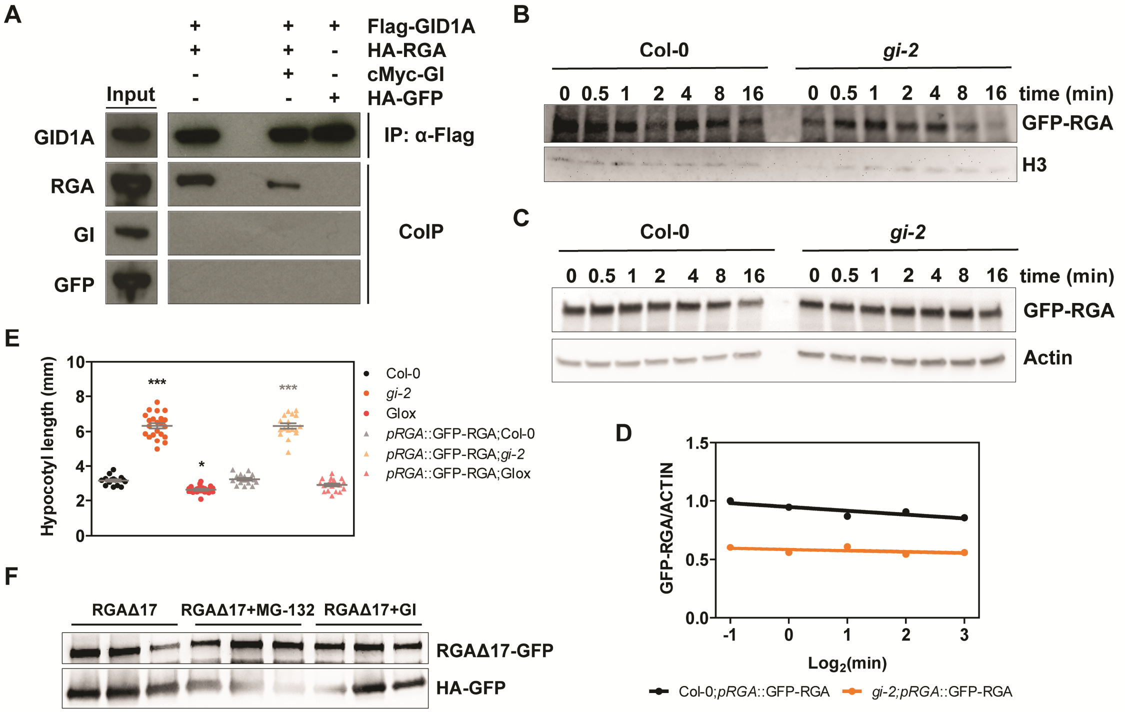

Fig. S3. GI stabilizes RGA in the context of its GA-GID1-mediated degradation.
(*A*) *In vitro* pull-down study of the interaction between Flag-GID1A and HA-RGA in the presence and absence of cMyc-GI. Proteins were expressed in a TnT *in vitro* expression system and immunoprecipitated with anti-Flag antibody. (*B*) Degradation time-course of GFP-RGA in WT (Col-0) and *gi-2* mutants incubated with 1 µM GA_4 and 200 µg/ml cyclohexamide. H3 levels are shown as reference. (*C*) Degradation time-course of GFP-RGA in WT (Col-0) and *gi-2* mutants. 10-day-old SD-grown seedlings were treated at ZT7 with 100 µM GA_3, 200 µg/ml cyclohexamide, and 50 µM MG-132. ACTIN levels were used for normalization. (*D*) Quantitation of the relative amount of GFP-RGA in every fraction from the experiment shown in (*C*). Protein levels were normalized against ACTIN levels. (*E*) Hypocotyl length measurements from *pRGA*::GFP-RGA lines in WT (Col-0), *gi-2*, and GIox backgrounds and their respective control lines without the transgene grown for 10 days in SDs (in gray, mean ± SEM, n=16-22; *p<0.05, ***p<0.001 Tukey's multiple comparison test). (*F*) Western blot showing the accumulation of RGAΔ17-GFP in *N. benthamiana* leaves treated with 25 µM MG-132 or in the presence of GI-HA. 3 biological replicates are shown per treatment. HA-GFP levels were used for normalization.

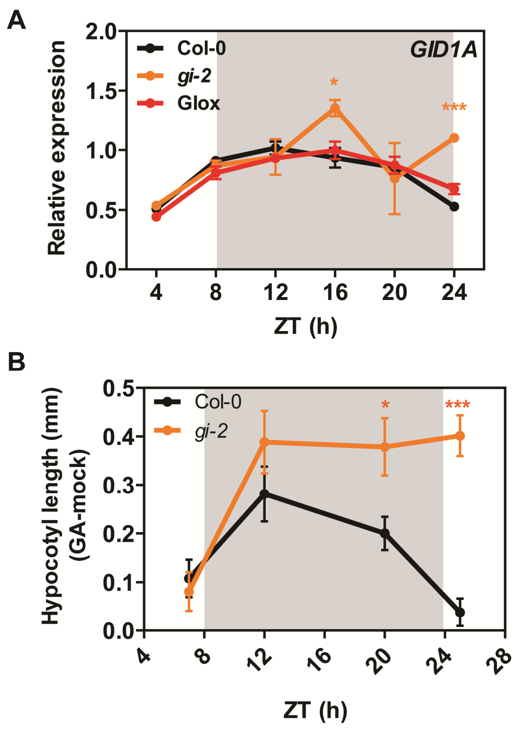

Fig. S4. GI is required to adequately gate GA signaling at night.

(*A*) Relative expression of *GID1A* in WT (Col-0), *gi-2*, and Glox across a SD photocycle in seedlings grown for 10 days in SDs (mean ± SEM of 3 biological replicates;***p<0.001, *p<0.05 Bonferroni post hoc test following two-way ANOVA). White and gray shadings represent day and night, respectively. (*B*) Hypocotyl length (measured as the difference between GA-treated and mock-treated seedlings) of seedlings grown for 6 days under SD conditions in the presence of 0.2 µM PAC and treated with 1 µM GA_4 at different ZTs (mean ± SEM, n=25) (n.s. not significant, *p<0.05, ***p<0.001 Bonferroni post hoc test following two-way ANOVA). Independent experimental repeat of the experiment shown in Fig. 4*C*.
